# Structure and sequence analyses of *Bacteroides* proteins BVU_4064 and BF1687 reveal presence of two novel predominantly-beta domains, predicted to be involved in lipid and cell surface interactions

**DOI:** 10.1186/s12859-014-0434-7

**Published:** 2015-01-16

**Authors:** Padmaja Natarajan, Marco Punta, Abhinav Kumar, Andrew P Yeh, Adam Godzik, L Aravind

**Affiliations:** Joint Center for Structural Genomics, San Diego, USA; Program on Bioinformatics and Systems Biology, Sanford-Burnham Medical Research Institute, La Jolla, CA USA; European Molecular Biology Laboratory, European Bioinformatics Institute, Wellcome Trust Genome Campus, Hinxton, Cambridgeshire, CB10 1SD UK; Stanford Synchrotron Radiation Lightsource, SLAC National Accelerator Laboratory, Menlo Park, CA 94025 USA; National Center for Biotechnology Information, National Library of Medicine, Building 38A, Bethesda, MD 20894 USA

**Keywords:** DUF3869, DUF3870, Domain of unknown function, Protein structure, Beta-sandwich, Membrane-associated protein, Transthyretin superfamily, Bacterial pore-forming toxins

## Abstract

**Background:**

N-terminal domains of BVU_4064 and BF1687 proteins from *Bacteroides vulgatus* and *Bacteroides fragilis* respectively are members of the Pfam family PF12985 (DUF3869). Proteins containing a domain from this family can be found in most *Bacteroides* species and, in large numbers, in all human gut microbiome samples. Both BVU_4064 and BF1687 proteins have a consensus lipobox motif implying they are anchored to the membrane, but their functions are otherwise unknown. The C-terminal half of BVU_4064 is assigned to protein family PF12986 (DUF3870); the equivalent part of BF1687 was unclassified.

**Results:**

Crystal structures of both BVU_4064 and BF1687 proteins, solved at the JCSG center, show strikingly similar three-dimensional structures. The main difference between the two is that the two domains in the BVU_4064 protein are connected by a short linker, as opposed to a longer insertion made of 4 helices placed linearly along with a strand that is added to the C-terminal domain in the BF1687 protein. The N-terminal domain in both proteins, corresponding to the PF12985 (DUF3869) domain is a β–sandwich with pre-albumin-like fold, found in many proteins belonging to the Transthyretin clan of Pfam. The structures of C-terminal domains of both proteins, corresponding to the PF12986 (DUF3870) domain in BVU_4064 protein and an unclassified domain in the BF1687 protein, show significant structural similarity to bacterial pore-forming toxins. A helix in this domain is in an analogous position to a loop connecting the second and third strands in the toxin structures, where this loop is implicated to play a role in the toxin insertion into the host cell membrane. The same helix also points to the groove between the N- and C-terminal domains that are loosely held together by hydrophobic and hydrogen bond interactions. The presence of several conserved residues in this region together with these structural determinants could make it a functionally important region in these proteins.

**Conclusions:**

Structural analysis of BVU_4064 and BF1687 points to possible roles in mediating multiple interactions on the cell-surface/extracellular matrix. In particular the N-terminal domain could be involved in adhesive interactions, the C-terminal domain and the inter-domain groove in lipid or carbohydrate interactions.

**Electronic supplementary material:**

The online version of this article (doi:10.1186/s12859-014-0434-7) contains supplementary material, which is available to authorized users.

## Background

Humans harbor complex bacterial communities in various body habitats such as skin, gut and oral cavities [1-5] that contribute to both health and development of diseases [6,7]. *B. fragilis* and *B. vulgatus* from the genus *Bacteroides* are among the most prevalent organisms of the human gut microbiome and constitute one of the largest bacterial contributions to the human fecal biomass [8]. Both organisms are part of the normal flora of healthy individuals and contribute to certain important physiological functions such as breakdown of complex polysaccharides in the food and nitrogen cycling in the gut [9]. However, they are also capable of being opportunistic pathogens causing a range of anaerobic infections such as peritonitis [10,11]. Both species, whose genomes have been completely sequenced (*B. fragilis*: [12], *B. vulgatus*: [13]), contain a large number of completely uncharacterized proteins, which are likely to play a role in microbiome-host interactions. In an ongoing effort to classify and characterize proteins repertoires of human microbiome bacteria, the Joint Center for Structural Genomics (JCSG; http://www.jcsg.org) has solved at high-resolution several structures of proteins belonging to *Bacteroides* protein families that are over-represented in human gut microbiome. We report here a detailed analysis of two novel protein structures from *B. vulgatus* and *B. fragilis*. These structures map to Pfam families PF12985 (DUF3869) and PF12986 (DUF3870) that were previously considered “domains of unknown function”. These are the first members of those families to be experimentally characterized.

## Results and discussion

### Structures of *Bacteroides* proteins BVU_4064 and BF1687

The crystal structures of the N-terminally truncated *Bacteroides* proteins BVU_4064 (*Bacteroides vulgatus* strain ATCC 8482, JCSG target ID: 393242, GenBank accession: YP_001301288.1, PDB code: 3kog) and BF1687 (*Bacteroides fragilis* strain NCTC 9343, JCSG target ID: 393243, Gene Bank accession: YP_211325.1, PDB code: 3g3l) have been determined to 1.85 Å and 2.2 Å resolution, using MAD and SAD phasing methods respectively as described in the Methods section.

**Figure 1:**
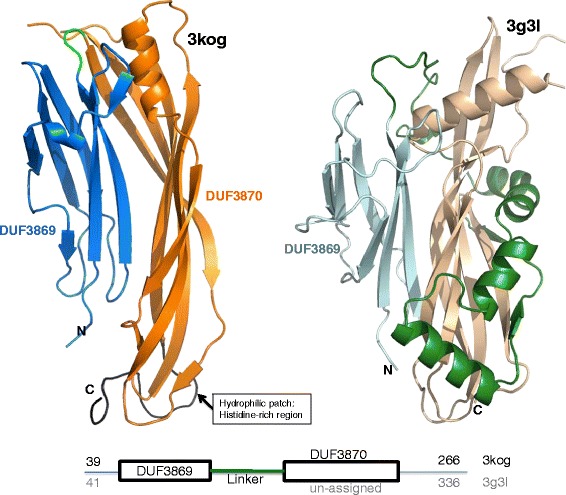
**Structures of the N-terminally truncated** ***Bacteroides*** **proteins BVU_4064 and BF1687 (PDB codes 3kog and 3g3l, respectively).** The N-terminal domain (in slate blue color) and the C-terminal domain (in orange color) of the 3kog structure show significant similarities with the corresponding domains of 3g3l structure (N and C terminal domains shown in pale cyan and wheat colors respectively). In contrast, the region connecting the domains (in green) is clearly different in the two structures: a short linker in 3kog, an extended 4-helix insertion and one extra strand that is added to the C-terminal domain in 3g3l. A histidine-rich region present at the C-terminus in both of our proteins is found ordered only in the 3kog structure (see box with text in the Figure).

**Figure 2:**
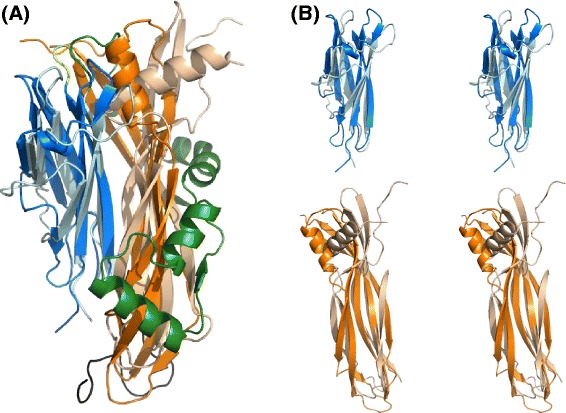
**Superposition of 3kog and 3g3l structures. (A)** Corresponding domains (colored in slate blue and pale cyan for N-terminal domains; orange and wheat for C-terminal domains; linker region in green) in the two structures superimpose fairly well with an overall RMSD of 3.7 Å for the 166 equivalent positions in the rigid-body alignment [15]. **(B)** Stereo view of N and C terminal domains shown separately with linker regions removed to highlight the structural similarity.

Despite a relatively low overall sequence identity of 24% (calculated using EMBOSS [14]; see Additional file 1), the two proteins are similar in structure. The structures of both BVU_4064 and BF1687 consist of two predominantly-beta domains (Figure 1). In BVU_4064 the two domains are connected by a short linker, while in BF1687 the region between the two domains, contains a 4-helix insertion and an extra strand stacking with the twisted β–sheet of the C-terminal domain; this region forms extensive contacts with the second domain. The N-terminal domains of BVU_4064 (residues 39–121) and BF1687 (residues 41–124) have an RMSD of 2.6 (2.6) Å for 77 (77) equivalent positions when performing a rigid (flexible) structural alignment (using FATCAT [15]; Figure 2). In comparison, the C-terminal domains (residues 124–253 and 209–336 in 3kog and 3g3l structures respectively) have lower (but still significant) structural similarity with RMSD of 3.1 (2.8) Å for 91 (97) equivalent positions.

An uncharacterized histidine-rich region can be found at the C-terminal regions of both proteins (shown in Additional file 1). This region is not ordered in the 3g3l structure, but in the 3kog structure folds into distinctive structure characterized by two successive “hammer-head”-like loops (Figure 1). These loops pack against other loops connecting the strands of the C-terminal domain, together forming a hydrophilic exposed patch.

### N-terminal domain (DUF3869)

The N-terminal domain of both BVU_4064 and BF1687 proteins form an 80-residue β-sandwich domain adopting a pre-albumin-like fold that is composed of a Greek-key motif (Figure 1). This domain is currently classified as an uncharacterized Pfam family PF12985 (DUF3869). Consistent with its observed structure, profile-profile comparisons using the HHpred algorithm [16] consistently detect distant relationships with β-sandwich fold domains such as immunoglobulins and cadherins for our proteins with the pre-albumin-like N-terminal domains. However, it should be noted that the version of β-sandwich found in these domains is a more abbreviated version with conserved core of six β-strands. Multiple sequence alignment for the PF12985 family shows the presence of Thr and Ser residues at −2 and −1 positions relative to a strongly conserved Cys residue, preceded by a hydrophobic signal-peptide-like sequence in the N-terminal region of the consensus sequence for the protein family (with details for our proteins presented in the Additional file 1). This motif is characteristic of a lipoprotein signal sequence [17,18] implicated in anchoring the proteins into the cell membranes via lipid covalently linked to the conserved cysteine.

From our comparative genomics study (Chang et al.: Adaptation of Human Gut Microbiota to its environment seen from the perspective of protein families (2014), *in preparation*) of the MetaHIT human gut microbiome analysis of 124 human subjects [19], the average ratio of the number of homologs in the MetaHIT human gut microbiome dataset versus those in UniProtKB [20] is about 0.07. Compared to this, the ratios for DUF3869 and DUF3870 are about 15.1 and 1.4, respectively, suggesting their significant overrepresentation in the gut microbiome. The data also shows β-sandwich folds comparable to the fold of the N-terminal domain in our proteins occur frequently in proteins from families specific to the human gut microbiome (data not shown here). A recent functional genomic study points to a large number of *Bacteroides* lipoproteins to carry an N-terminal β-propeller domain that may form an adhesion module [21]. Similarly β-sandwich fold domains play important roles in protein-protein, protein-carbohydrate and protein-lipid interactions (http://scop.mrc-lmb.cam.ac.uk/scop/; [22,23]). Hence, it is conceivable that these *Bacteroides* β-sandwich domains have a key role in the cytoadherence functions of these bacteria.

### Similar folds in other bacterial cell-adhesion proteins

**Figure 3:**
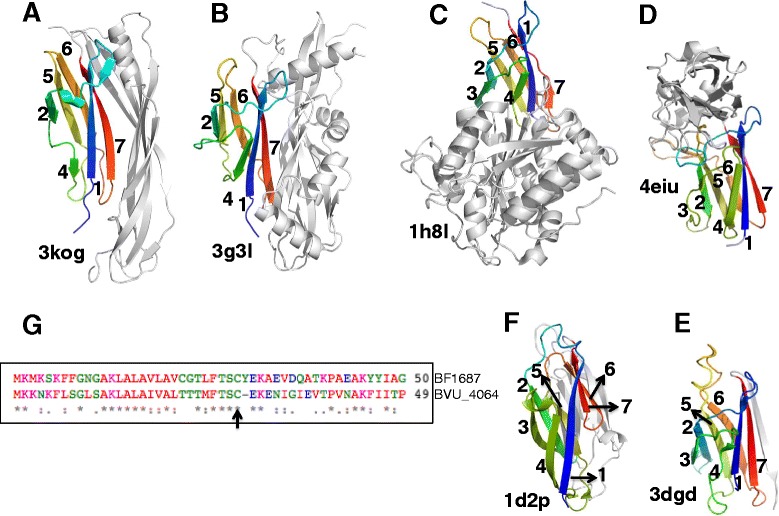
**Structural similarities of the N-terminal domains.** **(A-F)** Pre-albumin-like fold of the N-terminal domains in 3kog and 3g3l structures that is also present as a cell adhesion modules in several proteins belonging to the Transthyretin superfamily. **(G)** Alignment between the lipoprotein signal sequences present at the N-terminus of BVU_4064 and BF1687. The arrow points to the conserved CYS residue in the consensus sequence for the protein family PF12985.

Structural similarity search by Dali [24] for the N-terminal domains in our proteins identified several proteins in the Transthyretin superfamily (Figures 3A-F), such as: 1h8l from the CarboxypepD_reg family PF13620 (Z-score 6.2, 3.4 Å RMSD), 3kpt from the Cna_B family PF05738 (Z-score 4.1,2.8 Å RMSD; not shown in figure), 4eiu from the DUF3823 family PF12866 (Z-score 3.7, 2.6 Å RMSD), and 3dgd from the Transthyretin family PF00576 (Z-score of 4.7, 2.6 Å RMSD). The core of the domain common to these structures is a pre-albumin-like β-sandwich domain with four anti-parallel strands (3, 2, 5 and 6) in one sheet and strands 4,1 and 7 forming the second sheet. There are still significant differences between these structures, particularly in terms of the strands constituting the β-sandwich: for instance, the N-terminal domains in our proteins are missing the strand 3, while in the human Transthyretin structure (PDB code: 3dgd) an extra strand is present after the strand 7 and in the *Bacillus cereus* pilin structure (PDB code: 3kpt) a long loop with two short strands forming a β-hairpin is present between the strands 6 and 7. Yet another variation to this fold is seen in the repetitive B regions (PDB code: 1d2p) of the *Staphylococcus aureus* collagen binding protein (Cna-B family, PF05738), where the strand 6 is part of the β–sheet with strands 4, 1, and 7 of the pre-albumin fold (Figure 3F). A common theme between all the proteins with domains similar to the N-terminal domains in our proteins is that they act as cell-adhesion modules attached to a second domain with a distinct function, including enzymatic activity [25-27].

Supporting the structural similarities reported above, distant homology recognition programs such as HHpred [16] or FFAS [16,28], show statistically significant similarity between the family PF12985 (to which the N-terminal domains of our proteins belong) and the families PF12866 (E-value = 0.00054) and PF13620 (E-value = 0.0058) of Transthyretin clan, thus providing additional evidence that PF12985 might be unified into that clan.

### C-terminal domain

The C-terminal regions of both BVU_4064 and BF1687 form a beta-strand-rich structural domain (Figure 1). The BVU_4064 (PDB code: 3kog) region is classified as a Pfam family PF12986 (DUF3870) with the standard Pfam significance thresholds, while the BF1687 (3g3l) is not recognized by the Pfam HMM model. However, profile-profile comparison methods ([16,28]) confirm the distant relation of the C-terminal domain of the BF1687 protein and the PF12986 Pfam family. Not surprisingly, the structures of N-terminal domains in both proteins are significantly similar (2.6 Å RMSD with 19% sequence identity), but with significant differences in lengths and torsion in the corresponding strands (Figure 2). Structure based sequence alignment between the second domains using FATCAT [15] showed 3.1 Å RMSD with about 11% sequence identity.

**Figure 4:**
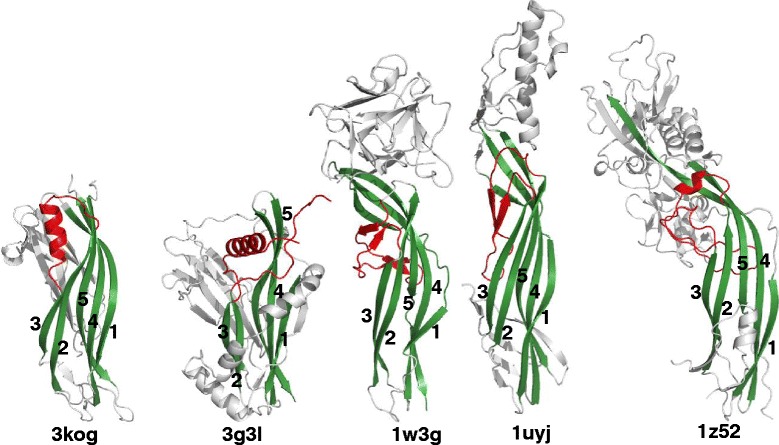
**Structural similarities of the C-terminal domain of 3kog and 3g3l with bacterial pore-forming toxins.** The region shown in red is implicated in membrane insertion in the pore-forming toxins [epsilon toxin (PDB code: 1uyj) and aerolysin (PDB code: 1z52)] and in the hemolytic lectin (PDB code 1w3g). In both 3kog and 3g3l this region corresponds to a helical insertion.

A structure-similarity search using the Dali server [24] identified the relationship of the C-terminal domain of BVU_4064 protein to the structures of hemolytic lectin from the mushroom *Laetiporus sulphureus* (PDB code 1w3g, Z = 5.9, RMSD 4.4 Å; [29]) and the bacterial β-pore-forming toxins, *Clostridium perfringens* epsilon toxin (PDB code: 1uyj, Z = 5.5, 3.8 Å RMSD; [30]) and aerolysin (PDB code: 1z52, Z = 4.4, 4.5 Å RMSD; [31]). Figure 4 highlights the structurally similar regions in similar colors. The C-terminal all-β structures are believed to play a role in forming pores that penetrate the cell membrane. A structural region (in between strands 2 and 3; shown in red in Figure 4) that is present in analogous positions in these toxin structures and the *Staphylococcus aureus* α-hemolysin structure (PDB codes: 1uyj, 1z52 and 7ahl respectively) has been implicated in membrane insertion ([31,32]). By comparison, this region corresponds to a helix-loop in our structures, as opposed a two-stranded sheet or long loop between strands 2 and 3 (Figure 4) observed in the toxin structures. The N-terminal domains in these toxins are neither homologous nor structurally similar to the N-terminal domains in our proteins. Further experimental characterization is needed to determine whether these structural similarities imply comparable lipid interaction functions for these *Bacteroides* proteins or if they interact with carbohydrates as suggested in the case of the lectin from *Laetiporus sulphureus*.

### Domain interfaces

Accessible surface areas for the N- and C-terminal domains in our proteins calculated using GETAREA [33] and inter-domain interactions computed with PIC (Protein Interactions Calculator) [34] show that the structure 3kog buries far less area at the inter-domain interface compared to 3g3l (1597 Å^2^ vs. 2362 Å^2^ respectively). The domain interface is fairly hydrophobic and is held loosely together by a few hydrophobic interactions and a number of weak to moderate hydrogen bonds (data not shown). While the residues from the C-terminal domain in the 3kog structure that are buried at the interface are mostly not conserved in the family (PF12986), about 40% of the buried residues of the N-terminal domain at this interface in both the 3kog and 3g3l structures are conserved in 70% or more of the sequences of the Pfam family they belong to (PF12985). This points to a key role for the interactions of the N-terminal domain for maintaining characteristic bilobal structure of these proteins. The computed molecular surface of 3kog (data not shown) reveals the presence of a deep groove lined by the hydrophobic residues (belonging to the N-terminal domain: Strand 1 region - F45, I46, I47,T48, V50, V51, I52, A54, T55, T56 and T58; Strand 7 region – L106, L107, A108, F111, A113, V116, T117, I118, I119 and L120; Figure 3) at the domain interface. As shown in Figure 3, the above-listed residues are spatially close together supporting the contention that they could form a potential interaction surface. Given their hydrophobicity, this groove could potentially accommodate a hydrophobic ligand, such as a lipid tail.

The two proteins, BVU_4064 and BF1687, despite considerable structural variations between their equivalent individual domains, have a domain interface, centered on the hydrogen bonded beta-sheet edges, that is largely conserved in the two structures. Thus, it is likely that they have descended from the same multi-domain ancestral protein rather than resulting from independent domain fusions. Their probable common origin is further supported by the observation of similar sequence motifs at both the N- and C-termini of these proteins that are shared by all the Bacteroides proteins with the PF12985/DUF3869 domain.

## Conclusions

Crystal structures of two proteins, BVU_4064 and BF1687, from *B. vulgatus* and *B. fragilis* species of the genus *Bacteroides* have been determined as part of the JCSG’s effort to carry out structure-based functional annotation of proteins that are part of the human gut microbiome. Structures of both proteins show a comparable bilobed structure with a two-domain architecture: an N-terminal DUF3869 domain of PF12985 family and a C-terminal domain characterized only in BVU_4064 protein as DUF3870 belonging to PF12986 family. Our analysis of these proteins based on sequence and structure comparisons suggests that the N-terminal domain might function as an extracellular adhesion or carbohydrate-interaction module that is linked to the bacterial membrane via a lipid anchor conjugated to the lipobox. This is consistent with similarities to proteins from the Pfam Transthyretin superfamily. As a result of this analysis, DUF3869 domain has been added to the Transthyretin clan in Pfam.

The C-terminal domain is structurally similar to bacterial pore-forming domain of toxins like *Clostridium perfringens* Epsilon and Aerolysin. It remains to be seen if these C-terminal domains might mediate interactions with lipids in the extracellular matrix of these *Bacteroides* species by themselves or via the inter-domain hydrophobic groove formed with the N-terminal domain. Finally, the histidine-rich C-terminal regions, present in both of our proteins, but is found ordered in one of them, resembles a hammer-head motif of the SET domains [35]. We speculate that this motif could mediate a specific interaction either with metals or charged moieties in the bacterial cell wall [36].

Preliminary evidence further strengthens the idea that the two proteins reported here are prototypical members of a substantial family of proteins that are widespread in the *Bacteroides* genus. Sequence profile analysis uncovers several additional lipobox-containing proteins from these organisms with comparable N-terminal Transthyretin-like domains (Additional file 2) and C-terminal histidine-rich segments. Analysis of their gene-neighborhoods suggests that they are often accompanied by genes coding for proteins with OmpA-like outer-membrane β-barrel domain and/or members of the DUF940 family of proteins, which are also predicted to be lipoproteins (Additional file 2). Interestingly, multiple genes coding for Transthyretin-like domain proteins of the PF12985 family might also occur clustered together in the genome of certain *Bacteroides* species (Additional file 2). It is conceivable that the OmpA domain proteins help in the trafficking of the proteins with Transthyretin-like domains or that all of them (i.e. the Transthyretin-like, DUF940 and the OmpA-like proteins) interact to form different types of membrane associated complexes. Similarly organized loci with clusters of genes coding for β-sandwich domain proteins have recently been implicated in interaction with and utilization of complex carbohydrates like xyloglucan by *Bacteroides* species [37]. In a similar vein, the structures and analysis reported here are likely to provide the models for a novel class of proteins utilized across the *Bacteroides* group of bacteria for their extracellular interactions.

## Methods

### Data collection, structure solution, refinement

For the structure with PDB code 3kog, multi-wavelength anomalous diffraction (MAD) data were collected to 1.90 Å resolution at wavelengths corresponding to high-energy remote, inflection, and peak of the Selenium edge at beam line BL11-1 at SSRL. For the structure with PDB code 3g3l, single anomalous diffraction (SAD) data were collected to 2.20 Å resolution at 0.97966 Å wavelength corresponding to the peak of the Selenium edge at beam line BL9-2 at SSRL. Both sets of data were collected using BLU-ICE [38], integrated using MOSFLM [39], and scaled by SCALA [40]. The Selenium substructures were determined by SHELXD [41] and refined by AUTOSHARP [42], which gave a figure of merit of 0.230 and 0.233 for 3kog and 3g3l respectively. The structures were traced using ARP/wARP [43]. The model building and refinement were carried out by COOT [44] and REFMAC [45]. Data collection and refinement statistics are summarized in the Additional file 3: Tables S1 (for PDB code: 3kog) and S2 (for PDB code: 3g3l).

### Validation and deposition

The QC server reports the stereochemical quality of the model using AutoDepInputTool [46], MolProbity [47], and PHENIX [48,49], the agreement between the atomic model and the data using Resolve [49], the agreement between the model and protein sequences using ClustalW [50], the ADP distribution using PHENIX, differences in Rcryst/Rfree and expected Rfree/Rcryst, and various other items including nomenclature issues, atom occupancies, consistency of NCS pairs, ligand interactions, special positions, presence of CIS-peptides, waters with no interactions, etc. using in-house scripts and analyzing refinement log file and PDB header. Protein quaternary structure analysis was carried out using the EBI-PISA server [51]. Atomic coordinates and experimental structure factors have been deposited in the PDB and are accessible under the codes 3kog and 3g3l.

### Gene neighborhood for PF12985

The gene neighborhoods were extracted using an in-house Perl script that scans the NCBI genome file for a query gi and determines the adjacent genes using the query (PF12985) as the anchor. Thus extracted neighbors (Additional file 2) were then clustered using the BlastClust program (http://www.ncbi.nlm.nih.gov/Ftp/).

### Gene neighborhood for PF12985

All the molecular structure visualizations presented in this report have been made using PyMOL [52].

## Additional files

Additional file 1:
**Amino acid sequences and sequence alignment for the two Bacteroides proteins BVU_4064 and BF1687.**
Additional file 2:
**Gene neighborhood and sequence profile analyses for the Transthyretin-like domain in the two Bacteroides proteins reported.**
Additional file 3:
**Table S1.** Crystallographic data and refinement statistics for the protein BVU_4064 (PDB code 3kog). Values in parentheses are for the highest resolution shell. **Table S2.** Crystallographic data and refinement statistics for the protein BF1687 (PDB code 3g3l). Values in parentheses are for the highest resolution shell.
