# Supplementary material for: Structure and sequence analyses of Bacteroides proteins BVU_4064 and BF1687 reveal presence of two novel predominantly-beta domains, predicted to be involved in lipid and cell surface interactions
Source: BMC Bioinformatics. 2015 Jan 16;16(1):7. doi: 10.1186/s12859-014-0434-7 (PMC4387736; doi:10.1186/s12859-014-0434-7)
Supplement: Additional file 1: — Amino acid sequences and sequence alignment for the two Bacteroides proteins BVU_4064 and BF1687. [file 12859_2014_434_MOESM1_ESM.docx]

1. Amino acid Sequence for BVU_4064 protein

>tr|A6L7K2| A6L7K2_BACV8 Uncharacterized protein OS=Bacteroides vulgatus (strain ATCC 8482 / DSM 1447 / NCTC 11154) GN=BVU_4064 PE=1 SV=1 (**3KOG**)

MKKNKFLSGLSAKLALAIVALTTTMFTSCEKENIGIEVTPVNAKFIITPVVIDATTGTDV

TQSAEISFSKGNGTYEGTPELASESININAKYKGMTGSASVTIPALKAGQFGAKEVTIIL

SENFFAQEESSNSQIETTKHSGFKNNTSDYWYYITVTYTKKEGSEVIKNDYEGDDSEIKN

IIDAYNKGVREDKVTLNDVQVLAHSRFSVFVDYMKTTSVYQIIEKSPKRDGNPVASFTVD

SYNTIVSPKNEQIPGHGHAPSHGHGHGHGDDSNAGGGIIIAD

2. Amino acid Sequence for BF1687 protein

>tr|Q5LER3|Q5LER3_BACFN Putative uncharacterized protein OS=Bacteroides fragilis (strain ATCC 25285 / NCTC 9343) GN=BF1687 PE=1 SV=1 (**3G3L**)

MKMKSKFFGNGAKLALAVLAVCGTLFTSCYEKAEVDQATKPAEAKYYIAGTITDATTGQE

LTTAKVTLGDKSVTSSFNEQVNYKAEGYALVVSADGYYPVKRQVYLNQVSDGQTSVATVN

VALVSVEAAVIPPVVPPTDPETDINEGEATKVADKAVEVAKPSESTVTDMLAGTTATPEE

KKALDETLEMAGGMKVGETTPEVLADGSILAITPVKFTNPIQDAPAMVPYFYNEGCELTG

DVKEVAAPVTRADGAVAADIQKAFLSNAAKALNMNAGFVQKIGYTRISVLNGYSILGYTI

KGQLVSKKLTFLISGKYYEGIVSYQKSVMIYPNYYSHDSHDSHDSHGFNPNAGGGSND

3. Sequence alignment (using the tool Needle at EBI) for BVU_4064 and BF1687

BVU_4064 1 MK-KNKFLSGLSAKLALAIVALTTTMF**TSC**-EKENIGIEVTPVNAKFIIT 48

|| |:||. |..||||||::|:..|:|||| ||..:.....|..||:.|.

BF1687 1 MKMKSKFF-GNGAKLALAVLAVCGTLF**TSC**YEKAEVDQATKPAEAKYYIA 49

BVU_4064 49 PVVIDATTGTDVT--------QSAEISFSKG-NGTYEGTPELAS------ 83

..:.|||||.::| :|...||::. |...||...:.|

BF1687 50 GTITDATTGQELTTAKVTLGDKSVTSSFNEQVNYKAEGYALVVSADGYYP 99

BVU_4064 84 --ESININAKYKGMTGSASVTI---------------------------- 103

..:.:|....|.|..|:|.:

BF1687 100 VKRQVYLNQVSDGQTSVATVNVALVSVEAAVIPPVVPPTDPETDINEGEA 149

BVU_406 104 -----PALKAGQFGAKEVTIILSENFFAQEESSNSQIETTKHSG------ 142

.|::..:.....||.:|: ...|..|...:..||.:.:|

BF1687 150 TKVADKAVEVAKPSESTVTDMLA-GTTATPEEKKALDETLEMAGGMKVGE 198

BVU_4064 143 ------------------FKNNTSD------YWYYITVTYTKKEGSEVIK 168

|.|...| |:| .||.|:

BF1687 199 TTPEVLADGSILAITPVKFTNPIQDAPAMVPYFY--------NEGCEL-- 238

BVU_4064 169 NDYEGDDSEIKNIIDAYNKGVRED--KVTLND-----------VQVLAHS 205

.||..|:...:...:..|..| |..|:: ||.:.::

BF1687 239 ---TGDVKEVAAPVTRADGAVAADIQKAFLSNAAKALNMNAGFVQKIGYT 285

BVU_4064 206 RFSVFVDY--MKTTSVYQIIEKSPKRDGNPVASFTVDS--YNTIVS-PKN 250

|.||...| :..|...|::.|. .:|.:.. |..||| .|:

BF1687 286 RISVLNGYSILGYTIKGQLVSKK--------LTFLISGKYYEGIVSYQKS 327

BVU_4064 251 EQI-PGHGHAPSHGHGHGHGDDSNAGGGIIIAD 282

..| |.:....||.....||.:.|||||...

BF1687 328 VMIYPNYYSHDSHDSHDSHGFNPNAGGGSND-- 358

N-terminus: Signal peptide with positively charged residues in blue, hydrophobic (mostly) uncharged region (underlined), followed by characteristic residues for lipobox and its conserved Cysteine residue shown in red.

N-terminus: Histidine-rich region in green and underlined.
