# Supplementary material for: Structure and sequence analyses of Bacteroides proteins BVU_4064 and BF1687 reveal presence of two novel predominantly-beta domains, predicted to be involved in lipid and cell surface interactions
Source: BMC Bioinformatics. 2015 Jan 16;16(1):7. doi: 10.1186/s12859-014-0434-7 (PMC4387736; doi:10.1186/s12859-014-0434-7)
Supplement: Additional file 2: — Gene neighborhood and sequence profile analyses for the Transthyretin-like domain in the two Bacteroides proteins reported. [file 12859_2014_434_MOESM2_ESM.docx]

The gene neighborhoods were extracted using an in-house Perl script that scans the NCBI genome file for a query gi and determines the adjacent genes using the query (PF12985) as the anchor. Thus extracted neighbors were then clustered using the BlastClust program (<http://www.ncbi.nlm.nih.gov/Ftp/>).

Selected gene neighborhoods with DUF940 and OmpA domain genes

---------------------------------------

ORGANISM Bacteroides fragilis str. 1007-1-F #6 accession no is 695524080 gi is 695336271

. cds dir len gi gene locus pid product

. 166269..167516 + 415 492242025 . M149_RS12875 WP_005786554.1 DUF940

--> 167527..168603 + 358 695336271 . M149_RS12870 WP_032533915.1 PF12986

. 168671..169420 + 249 492287628 . M149_RS12865 WP_005799579.1 DUF940

. 169482..170600 + 372 492242036 . M149_RS12860 WP_005786560.1 OmpA domain protein

---------------------------------------

ORGANISM Bacteroides fragilis str. 3988 T1 accession no is 695589623 gi is 492242036

. cds dir len gi gene locus pid product

. 7..1254 + 415 492292077 . M084_RS14790 WP_005800616.1 DUF940

. 1265..2341 + 358 547946568 . M084_RS14785 WP_022347567.1 PF12986

. 2409..3158 + 249 492292075 . M084_RS14780 WP_005800614.1 DUF940

--> 3220..4338 + 372 492242036 . M084_RS14775 WP_005786560.1 PF12986

---------------------------------------

ORGANISM Aquimarina macrocephali JAMB N27 accession no is 639967002 gi is 639966967

. cds dir len gi gene locus pid product

--> 162418..164739 + 773 639966967 . Z054_RS0107875 WP_024769536.1 PF12986

. 164754..165596 + 280 639966972 . Z054_RS0107880 WP_024769537.1 Transthyretin-like domain

protein

---------------------------------------

A profile of the Transthyretin-like domain based on 3G3L homologs was constructed using the PSI-BLAST-in_msa option and run against the non-redundant (nr) protein sequences database to identify further homologs. The list below is the set of significant hits detected after 3 iterations were run with this profile using an inclusion threshold of 0.001.

List of Genbank gis with Transthyretin-like domain proteins predominantly from Bacteroides group of bacteria

695336271

695493967

492242029

695438301

496043806

695553033

492268824

695502534

595997144

492292076

695486380

595978820

695399468

496602039

492204738

224036439

492356594

547946568

695472490

665397283

640565501

551208946

696209764

700260288

700234434

700246161

700225552

700218812

517795711

700224493

696215214

545303831

545425856

564601814

492521571

545442288

545441383

545424155

545440091

545427710

501433055

651338296

503582319

494156154

693557768

693562450

490722656

496417107

647201394

671546886

610629893

496520837

670495195

700535013

647519172

503071911

639966967

516846372

667812483

537743599

497865946

502690398

497464727

517449762

653169929

522094720

506375089

652743696

653170649

652870585

503761747

496648199

695486064

495936365

695486160

695335756

695440227

695474195

503991059

695492400

647514206

492247218

499516505

695432192

640642812

695590030

695582295

515150116

522022178

490515449

596226503

492220931

695345778

547278854

695338361

657688770

492361013

492302997

647298776

596254907

695422210

596136953

595962514

496326324

595895778

595889473

596144604

595995569

491882595

595908338

596130255

695578688

695341781

596122096

695513654

596098830

695516285

695343966

491864807

546445284

503433366

491875097

503800667

695524185

503174767

687223064

522055539

495737572

497269292

503968325

652313007

551201160

695502037

503787790

576321836

653145968

652596215

695580485

671545083

652200982

695341943

495547144

515557836

648568165

671231067

700543544

559212850

500933359

647296565

610629579

568802589

495079469

517443496

496578990

692213473

547896712

498221620

496042783

499421118

488789200

488742790

488787483

652625791

517284495

652303692

511014658

696274017

330752449

548252898

298261106

652307047

512457958

545386841

640829926

498348438

668937890

506288594

652374221

495430289

497432307

497326464

596176491

559193166

610421530

496581393

596107800

690717158

631783176

576320809

651338414

522085130

598873655

503533356

490259344

559200135

658508696

503850297

495426344

652377171

652509394

647330448

652510247

516064853

652327755

690714626

655257702

149355371

652331737

671221092

494838125

655074195

527072581

654408101

516343311

658508701

652599303

640722182

491929545

503397631

652593183

652359674

547310665

496585232

507740214

669228055

696234512

651695795

657024909

522019214

575462562

610625495

504841660

495532413

503931238

658508953

494925644

653144327

684211641

696366668

640550549

500639852

524326167

507754189

503757326

517459428

688464407

654414247

647197508

503517179

503634589

515827477

570746386

495914657

489077059

503314214

505079240

497465772

502694390

639969177

495516283

652442906

654838644

502606487

575457639

655160478

495889521

695125096

494229443

544632713

655910494

503832669

503761238

657039486

655253617

570741863

518787503

503384939

506293246

517447993

522026359

517451940

490422030

85816700

498203822

655490398

584428811

651360728

503985185

489101739

670496598

518218165

647587932

658502011

490705304

670505902

510821531

654602311

497938759

651327301

503693486

502457070

495430291

559197414

506295324

491859541

657031866

522094575

503209316

647295831

693567492

488792574

495426340

655382096

503432726

506235604

495514027

647196425

697008459

506234681

493987256

547310795

700266122

652381416

491929522

547918915

652296390

653073801

491852403

495429539

494046158

504593942

648501945

547919533

503696443

647590065

496057752

492463951

491876239

503788910

518500081

500644427

515827394

502606099

517982014

527065560

640587845

568803418

503697241

212665029

671231065

696360905

495781501

493895810

489082798

652758783

515147446

496943330

504587959

725817544

496279428

498212114

524327608

671238437

502452969

491934698

503787210

652618771

511020512

652419840

505732148

503992101

493848670

498499902

651338514

489084771

662568539

495781776

519058185

488792855

491939156

495422031

503984463

503432811

652559305

670480520

700535056

723442849

652415109

503966675

317388355

497545457

495941931

610625920

489096608

503990398

653169931

693554732

497284891

498107224

653175849

499903286

646943325

647197756

294448544

496957236

498209286

695528980

522057445

495431398

639135879

495422126

491872796

658508567

696363847

503383698

667025371

652851474

502952721

503797898

498286219

684138501

654925220

522018986

524326227

657032298

492454799

548318967

660631781

488737651

694993633

651361458

657640574

657645239

640590214

652540817

651340232

639970666

504186251

510936743

505147470

504612371

505293538

522018319

518834778

646874750

665891726

496413630

503986420

649530285

583252132

674968524

654844724

547252278

696358449

495123127

496055807

654830171

670477954

495116800

671763186

496325900

496047278

503174812

495929553

496674184

512705895

492469922

503219037

392628984

488733033

497870387

547252438

496575243

491933055

502457468

546450162

655321844

496523478

488624937

506262905

505078360

651694484

678232131

693571803

500656902

492493684

503967091

497913573

671713916

547774114

495430357

678228550

653073661

506375043

491610022

647207107

500027812

498214342

547780980

548783285

502837339

229454050

547311538

648518966

503534785

495116075

517286977

497436455

496288951

648641962

660632158

545407934

496950138

149129858

655317659

653146024

522058766

653275641

495931416

496278647

649526724

655992249

517498237

695551113

495291858

648635300

496487339

695473261

695470717

496054270

695471642

503218792

695508533

695407126

695527014

695523991

546361592

695562565

695511819

595911147

495426256

653059960

655256377

695397705

492296458

695436570

695471234

491882634

596026911

695496253

596139657

695330847

518368979

547376158

292636499

595965225

695461172

491875137

495933765

488747718

695485234

697009755

653175961

482610024

596080145

695521542

695341628

695513197

497865781

695428578

695418872

492336862

517286352

496963875

596124663

696260377

695483927

695516900

495889860

495111661

610625360

671542557

671543494

517284541

522018661

695334920

499515416

695445117

494931306

695492256

665399131

495947873

492276072

498501583

492236896

496422519

695500959

491872467

506291116

505173704

576505584

493897084

494849417

640564392

640554430

493796858

503383633

652611237

695537271

262356926

495785926

547780870

496417086

503788812

671234229

490439721

517090750

548768256

492502688

491935040

671715595

544899891

492703045

676328045

495116623

672714104

660629898

490430298

548317778

505077308

547920186

495115766

696358993

503311260

495109033

671713822

696267258

695394469

547774330

495298431

495126142

494929201

495131228

492740928

496280478

492357731

516737427

696276004

522017103

496600721

503219316

492478787

498351297

695481061

655994997

229451814

652743491

655912215

647329019

547309852

695497965

495430082

696279592

495424309

503211559

491882792

547789132

263254593

495129883

502838736

649507104

517263096

490430374

501003052

695342401

492213099

547279219

491856988

695345588

695333750

655319807

660633157

517455436

496054667

649550828

488754228

494747755

496329757

547774196

646871187

492337630

488750414

496326161

488621811

503787670

500655478

545408018

517442703

298515213

649559897

595967816

548238585

492501551

490437768

491882809

595940335

671230683

656326085

496049737

490430155

490452998

491875391

292636170

494744124

656326181

649533591

497546229

503757877

511014668

496575054

696368128

652559612

547781644

498500445

505080631

659418292

490452258

496055092

651641890

492477062

517283059

499517038

229456661

494938195

647328596

695469974

695331496

499302962

503785281

695442240

695128995

544899553

695576537

492498564

671226280

496573818

505080184

639979646

504838092

490424258

496307963

495918019

695493838

496952614

680467812

596203616

494940512

596253518

695508230

496648072

654843938

495421811

496045630

695433983

492279683

596187916

696357400

567444017

695404540

547825282

503764199

695468820

595887861

496476968

596135684

659415925

655077819

671542581

492320258

492227648

670479916

725817565

655076166

655119416

511017379

495890796

547233854

596002307

491851878

505344650

504200897

503789012

548318437

547195713

692211976

655448343

695516845

492254911

494926478

522014970

696371284

494928864

671211769

595913761

640588961

695519504

695456973

496963964

503452197

499228080

496326171

504065588

495936950

503787890

495120467

495117762

510936615

495512412

695487237

547277462

648274056

492365372

507737908

649552952

695496361

695334017

695342800

695467278

695510921

695332975

497268728

695342251

695336550

696234855

496054805

695479064

660632870

492228219

496928393

496307691

510869658

655992807

595960530

492502061

498500379

492498552

502466376

567449687

229450979

496965295

545407485

596120278

653067912

688464064

491880808

547231581

648623166

518369648

610422180

545406299

496488043

527057546

695578995

656214838

506291816

511021221

492439609

522026713

653564307

492455047

495431567

495949046

649547635

567410134

496573815

496056838

655321594

504595528

496326142

496421957

494742824

654845390

497545918

503384222

517980278

496138530

524325927

546448761

298260362

696378164

671716274

647470685

495115700

491872190

506262711

515149976

654604401

496936069

496421323

652560037

695573570

506294416

696274177

548170282

671710338

655320193

488752961

695564536

492267577

659855484

695518783

652414321

496416121

695480353

496578707

690715424

495906247

657617404

496956234

695331401

496582117

496950613

335939831

503430275

649533582

496044233

695439228

495024079

547869395

496048322

500657861

503990605

723444636

548171650

494750346

512709036

488752069

496927777

503534112

696370108

547946421

567444501

696273210

695466318

495928126

494406510

494607222

695494478

695400667

495128022

503384224

495112781

653250180

504843843

548236308

651643308

493852239

659415232

655319038

567410445

495416477

695503552

504064909

492703277

547947083

652358768

492414637

488625839

517290124

702958994

496307796

696378667

499127713

527059454

499516146

651339108

649565994

496049733

503759688

496946059

547780976

517455174

695508684

499724706

512709441

496453315

576502438

494744173

495933947

649506989

647586866

567459258

547871733

502607175

695556736

655257136

544899804

492477074

659416160

695335528

495431331

488621963

548181033

488625540

695486505

653243833

490446308

503174414

670934940

647591323

657714670

659418297

695339894

524326183

548254532

647299431

649553235

671553005

498501999

695485615

695505986

695516057

649520134

495121162

655320240

503219275

294448327

547316090

596134475

496055089

492198297

671712469

695559690

502607498

496038405

506289718

494413388

495114643

494406708

647281096

503429147

658501719

695536743

652416850

652355802

499128113

640589363

492325047

657617362

671546849

547946611

499421294

496575569

496486539

567762208

547800328

649557472

499421426

547278356

671714109

496429117

547953578

503788892

496278488

499124782

251839075

695450405

695330955

696371326

655318008

696363921

547252448

492307923

655318165

657661681

527063818

547282521

490452970

547195182

495935605

512707037

568803433

496954461

496329721

647467473

651694388

648635654

695481671

492299946

696263993

695540312

695337493

504065353

690716916

502447094

492280945

489094128

695492841

695575776

492238343

496452840

499125614

503787624

503525511

695429271

492303862

488625477

495939876

695495798

695486774

499301161

504840174

510823167

692208336

489091374

649573388

652367419

655257097

670494120

260620653

494932165

649567764

648658988

502417992

492385986

511023468

496574239

547798581

490452206

490424308

497437358

495037374

695590479

595936387

502827900

551197058

496487976

547870053

573454116

547952848

695551891

695482495

503787659

547683662

496049320

653123230

652552391

502606388

494751533

392642341

727805881

491867609

511023719

567457403

511023717

498286182

492349722

492245597

647587826

317387981

511015511

492741921

489102365

695431248

695501562

488788569

568802567

518784832

695527601

499124129

596031058

692214094

496412622

547897173

494400390

517869375

498499929

498500685

496601224

491856008

596215770

512184074

499302154

488752871

595934527

659886574

499515579

567764960

495934207

551202675

493852053

547251620

506294403

489070233

496279640

596018330

492690687

547926390

490424737

492717891

506261978

596267808

697061150

495923780

494744906

696376668

496579333

654926841

595961673

595897018

522094781

496579336

499123872

490420029

655518692

653248918

640576440

596109388

653074421

657643073

547939396

547897533

596123012

595971898

547323403

517457269

491856942

652744214

576505424

647281151

496522649

595969777

490424105

576321886

652520737

522022968

647518532

647585493

651694879

695445437

492736610

657646013

495906659

655994625

494401540

671712922

499124765

496960425

496486688

547919318

491884422

547282902

653565030

490502181

694993491

490461200

496329174

695421015

670934760

504841372

229451755

492449663

659417037

596043187

595932711

527060255

496423575

652520814

500646811

548171937

492375281

647518369

495033593

595978271

500644431

570745610

511015756

649545097

547810399

492273045

647545251

596233634

657617831

490430877

595973642

547714439

517091381

496050414

504840337

596280810

495474415

491875282

263253291

596129581

491875649

522084918

596214208

596034580

499124502

551207833

648239658

655253717

495419883

595963386

595981544

648570687

659855700

674271717

496530093

671715576

654844778

548179476

596188734

545404573

697092091

524326173

596275488

522017424

655381724

657614255

655516185

490434908

497545600

503694117

492479632

492430335

492408956

653051296

492404727

492391843

499125257

573502818

659858002

502825836

646947017

635635159

498500799

567761428

700531131

522090451

496964394

503757682

703350810

655522225

495947627

494935750

647558932

492251820

495428134

495932927

655321974

503430542

655322113

504495233

506288689

505081234

503311763

495128674

695340294

595939044

695461127

640561149

492237432

492275975

498500695

527057584

652503883

695422997

490425936

492502518

695586979

494928695

498500348

497546108

651360564

697057612

498221596

495429506

649553667

490438874

545304676

596046574

697059395

649552402

498205832

495422584

503761065

547774057

659859509

688465934

695481140

496280436

547309559

695515809

653129232

491931446

503532188

548181000

488757392

547788695

657661534

495917118

496331788

695551158

655318750

548254180

547286633

494746718

586958055

695345042

496421425

547186086

695334263

498500396

649544795

511019029

492410391

495939149

492382618

505146937

695339539

659857753

596171058

500656512

518790734

547789612

596137959

649551244

392687399

671713283

504840597

610421744

495299121

491882399

490439800

695334291

547946674

496327967

511019778

655319565

490454715

492371507

496279459

695433418

497546170

496582731

498500009

494929674

648518913

392639244

496055454

494747652

671715563

496286722

517285989

503788676

496529847

527059475

495948003

490455249

505144257

503432310

647328077

492439867

503967093

495300889

495122048

496051617

511022412

497546561

647280772

503758221

654407558

647589866

517796951

495429908

503313361

657214090

647553382

537742132

404575366

495118803

496476792

647587987

492482856

495130640

517289129

648544220

510869653

500646638

503759942

496574857

505345703

518501212

496331715

498347964

496418447

544900670

494934034

648518524

498205276

547869514

493895606

518370748

490442868

496420301

522089615

506291361

494849475

496579208

703183436

498500386

649528624

502837626

547308550

522021103

496047614

495106072

502828660

495123473

500654456

518784846

493850910

495114207

502459382

651361217

547331605

649530098

492473345

503784244

491864557

503832180

657640428

655522993

490471723

497546156

547278089

404585794

547810463

511013583

495945029

496416459

504065406

491882096

492254717

492281258

696372511

646934563

651643808

495521220

507740553

696274115

502695611

695506479

695493483

494400575

499419694

498223323

496035999

695481614

695523033

695457865

657643314

651642232

695488557

492349686

496328636

345454491

547789934

695556993

648664598

496049202

493326018

652510715

496043398

495112038

229456680

499420429

511012647

494823364

505077155

495933105

547781267

498499277

647544017

492401559

505145126

503758778

547788972

492488329

493896430

648617914

522018595

522021847

660632687

652870415

671712914

496574461

545305115

498501213

548316786

492476928

494401549

491886666

567764550

505345082

511018415

660634443

548253201

298260644

498500000

490427772

495128634

671543527

544634011

695492846

687222709

496039449

490425507

492406534

494397649

639133497

492401347

547234080

495131244

495115730

695479002

517451503

495887699

524329386

495925137

494153641

490424027

495114713

696273170

511021125

494929022

696268087

495299950

503217633

610421569

495841527

696358889

547774338

652562076

697632338

494418352

495121207

497869966

649573422

511019604

494932107

496522445

490452414

506294989

657616840

640588257

647280741

518426885

495916652

494926640

392634984

647587747

695334055

495928405

518785502

382948303

149128844

496574458

547185209

671715939

547799761

649557732

496575249

504065441

547708979

510936758

497947828

657662233

488621757

495111734

595911412

392694724

657032581

653275910

596178490

595985080

670478335

596101869

500657696

491878225

640650605

495128381

496045478

499302771

570745017

696277300

496484969

495520400

492276237

640827838

495428597

643431598

648502107

657617482

492482829

652763635

335942941

651643358

696277753

659418232

503990777

313137786

503785309

504064196

494306347

492299513

496044750

654925074

647607487

663455630

695513574

649534514

496508639

652560885

406884187

596027001

695514681

496600602

517498015

496278459

527059462

492740152

596219706

596199534

596091572

595994570

595969258

595906223

492695301

547774488

547670528

596085898

494653054

596042098

229443494

518788002

496055813

496055444

695526824

547782236

596118782

490475788

547780885

492502709

492236495

382948812

496047288

695343607

595947939

503398550

653066781

695471686

492295758

695451864

489089699

660632193

511018667

254835546

696259031

496285738

547230461

503314612

649560166

548153494

496288153

649551137

500655142

495924396

548171332

695513899

492238347

504064324

654482058

546359692

659417047

649563178

649531557

491876202

495432141

663457500

680467100

504586596

492426248

495120768

495114772

660633755

488621713

495119952

547749109

595961449

695527615

649530087

500028893

503833288

695437235

695543919

695333309

503695736

495021728

660632933

517456766

662332681

260621677

490449208

567226172

652552935

1100065

496048576

696234563

548317345

548172526

651694472

696364281

497466492

524325417

559203124

696271594

596050445

495515694

502838629

527062924

495042299

695423328

695450696

646883026

491937906

494399702

596283419

494747609

547322230

670495556

696262255

647552158

649509077

503797907

506290516

671214666

392675453

695488425

695403504

494418221

671546894

547715712

647329859

527061690

547746176

496040635

547715247

292633775

639136979

653565060

496601226

647281265

503787886

697058804

649503757

547799258

492469952

495915855

695126967

298274042

496330438

492394934

596029278

596002799

598868555

495085953

696267616

490453417

596076360

511020569

491939203

547954218

313136573

495945811

492464193

494928497

688466189

488623399

497546583

507741185

298263641

495036834

700531312

503433352

595907259

488625527

653074682

671714414

696361254

503379106

695520028

503311372

648443872

496475305

229455864

511022643

652577970

527061168

522084924

595940852

494413910

517155345

503757468

498499539

696272244

647330154

492369351

696367794

635635756

648542671

670936962

518370739

695531042

651733599

696378846

547780855

482605175

663466355

261868889

496413038

695331009

492254207

496308630

495123736

495126209

496307995

659859429

492349588

505077161

490515597

492444848

547323216

696279180

229450737

496422629

548169970

503762902

495296906

696378832

596204578

695522994

655994958

547939000

648570373

495944934

495131332

496573854

695339500

671552452

518834219

527060117

499300991

503692119

547774223

505146546

547879125

596222856

547845232

492329759

546448170

503315084

505344372

495108270

547190107

595898531

695489135

655321929

518834562

547798854

492736956

490433140

696365928

517498154

671547138

517284947

505077036

262355783

655321191

655321654

595984471

697985414

496930448

506291306

547283127

547199011

229450888

652871156

494417350

505345212

703349119

494749368

655517731

596054532

547798448

504491900

695489606

655321748

491938014

505345712

658508427

547809975

392676840

382949431

408471925

695496926

503987531

492439120

335935946

596014259

695495834

544900335

522084904

596241275

695331283

695510204

491934690

229449610

495296987

695470789

495131478

595940994

494935192

498501436

695501927

498222795

596152137

503382929

229456701

522095270

298271455

490423866

495297015

496055131

490478451

596029421

491866317

496098776

511023103

496278426

649542453

663482188

506291147

586955393

648518844

695516647

495126186

503634977

548237263

696364002

649528492

496300335

490427734

655321736

658643678

488622141

649518976

298514746

695436165

653563766

548170466

696231097

596023204

490475083

548253966

488622651

545407456

695586709

695411058

696233843

695346250

499516849

695331192

494223210

655076928

500646770

695516746

500656660

696273668

496055416

696275476

595932620

595895000

647452724

596002913

695433213

649548030

495949875

651340268

495118572

490469649

490517384

212663678

676330527

596007892

496285852

499515271

695470924

496308813

695470688

695346133

492324927

496929890

546187591

501002758

345456183

496418120

653051473

695436318

695336899

652485597

491859356

696273073

649521312

496416612

507744058

649502518

678225475

548236512

640642597

527066191

503757663

544900307

695464214

695293987

649556993

502692504

292636589

298271263

655256147

596136401

596091622

695478483

695346186

499301047

492219174

503531888

502466378

695570498

695537141

492329781

496423545

527062890

547190020

511022212

547330942

696267240

695460618

695454965

695295002

696272234

649561859

649557075

547788481

518429917

496328487

497870063

695342769

149130092

522088463

522018853

696273187

680467107

652869790

544900532

495431604

492369338

492236471

504064195

652510787

547920656

496574437

517263325

499515329

497546371

547309627

647617750

697984574

492464883

494653645

596199444

496044748

695340067

496411358

491869614

649565760

547310248

655521416

649510355

492276250

517447247

527059502

652414384

491931870

647510415

524329697

496474884

262353790

496307641

524329024

496582813

496330868

547278789

492219462

490453789

496416908

502692506

510821116

502690075

696373115

335946501

517448347

696363827

490480223

490418289

576504908

495422252

505344774

576500839

695571229

496600231

655257135

496054215

504586603

495293604

490451287

503788661

584444759

292636376

496579666

524325837

495475465

495429958

548171814

696276351

524326257

492737081

654481520

655318917

596181231

522084992

576323841

492501461

649531256

547321722

547198568

503831984

595910709

511024014

647436138

573501772

490419616

502605692

570745615

522020012

490420318

505078022

696278907

511022741

695334833

292633354

505345961

495299777

490421005

503789054

655119724

494399836

648600701

335941486

491851804

500656531

482605919

504840969

502825711

646936693

657925383

498500565

392692830

500657834

492295899

504492711

547230560

496414655

596124403

496055128

668934487

499421755

648539732

649544994

651360977

653144837

659855122

517868851

505358905

491875352

659414992

696371477

548316652

517961199

548171756

503430509

649573145

647553148

649532607

648518491

547906061

654845778

491872425

654925686

596098068

495900542

496329840

659414903

495299234

567451276

503787860

695513831

497822979

298514524

229450037

491925176

657615253

494411666

639134048

648539681

294447942

491938779

595904630

499128108

511018076

494846882

655318308

498203304

547919049

547920452

595998763

695587443

490440078

495943808

392692262

229442540

497546013

595961592

548179748

492406304

696279765

696264926

655520837

696270913

495906620

547781534

696276879

547788949

298272151

696273144

576323043

488749780

490424922

505080958

696275845

654845405

503531830

503787820

260621133

655548842

696276370

547667063

647205482

547780850

654845175

670477073

499126910

498500403

490451416

490415826

514973156

496042497

547946633

492295648

495917831

651340901

527067164

499124401

670479545

511022783

494939110

392623934

499421474

596033110

695492923

695336621

488758200

647326958

696232215

313134251

490429422

335933449

659855371

610422413

596153184

596020481

655522553

696267703

503528994

649509979

696266375

499419754

695504887

695471772

504064341

690778323

496044703

492482449

492491592

495022978

687221646

335937873

495926174

595899321

703427505

649560300

496307992

492476921

494934098

547257448

262354269

696274891

696231223

596223691

595911460

404581930

695544592

695537843

492238532

495034652

695341512

491890021

655549439

496420303

517290750

503984630

660630183

498501502

640718871

548318984

503788710

596140339

596125148

335944942

404577862

491880798

692209863

610625577

596061127

495427733

595992753

596120054

494928466

491863056

494224409

696270806

649503016

547799111

696260664

651328304

696371012

494829375

496050912

229448843

547781481

492496489

517089402

654924841

494400472

511024197

496949500

695330876

657616738

655321999

494406605

482609711

596217803

494399882

693568154

263234372

548171155

491875357

298515580

695479161

695506131

652574993

499722220

596039226

495839802

695429328

492498657

499301403

653249087

547789579

671543743

496039716

494416382

547278970

648518595

490434665

262356685

292637825

496040016

695532355

492320516

495917627

490438118

517979240

655992180

695557889

492279515

596147508

596085193

498223500

491865791

653243517

382973794

504584856

496054578

496579004

692212297

649572920

648293948

695571511

696273009

670495409

490430523

586954967

547798683

490426436

505144169

547310064

546453311

660632508

649516796

491939585

491932907

522021922

491867627

496345954

503799605

547279262

496331560

547954471

595974376

547198225

655073900

490477677

694992408

546841205

648635573

595936815

490444691

547251835

547323385

491891039

496574137

647543455

503692555

503430288

657618265

492276376

695486567

547946872

492255814

492304855

649562722

498206828

695489329

492455160

544900398

655074465

491883435

518812083

496040955

503787574

493986191

490454082

696275055

696231230

670938412

503759584

695485028

522096392

648592901

596033117

496049993

495478375

495301695

695409837

517089502

657641008

659855164

640641668

652561024

598871070

382948319

652743279

547781922

496041609

548168476

494833291

495427975

511013480

547897097

695570338

655073568

503759499

493852788

695526600

496056299

567457246

503210673

503315505

696269068

695496146

695516779

506295254

499420569

547896741

495474666

517284562

653143001

511015284

490452052

496036144

404580197

524325556

495518999

503757557

490416863

492482151

647471910

490433668

510869262

696272218

696264765

505077963

522023058

503785446

696279579

595914740

496932007

695486045

503787899

503992219

595892690

496307554

505143677

496049997

496422401

229457330

596039356

518428713

657614641

653184325

495512263

595925469

519055853

495425965

496307412

692200377

506290458

517288320

652618023

727805622

503532036

547198548

503991514

635634798

494834870

490421832

647589838

522015682

649556249

548172079

695423104

517290656

502692081

546361553

696274006

335940990

491929514

496346145

292634299

655521374

495106633

496053241

596134285

649559558

565960139

695482348

294448683

547782226

492482875

506234821

498225248

696232182

568803356

492450119

657615277

647548154

647617992

524327109

503529236

496049225

495433690

522015738

647545669

511023460

495515285

495511527

595984411

551207266

693560566

654602863

695455794

503691991

596253298

595969679

496053659

547714026

647510220

494838489

496453512

696275691

596108595

292640054

495513254

495422190

490420687

576499955

544900308

596176618

595980927

595940233

499303003

494940514

298515518

494743665

695454248

547824026

659415006

696267311

491865647

522018568

649504372

547897401

697058824

671712509

496048379

595997787

496530694

496287771

511022912

500929589

494010758

695330391

596094852

503785444

502451233

503220643

492478075

654843324

649532678

492699573

695455501

544900818

648570230

495431287

498205851

696276187

598894708

490418217

494838374

696276184

596254809

596135002

496417633

598877264

649528315

547231844

547801131

494413754

596154340

504494795

490421777

495887821

518428076

671709430

647326236

294448869

492406528

503174305

695339471

696272220

547310937

595911521

490509438

335942885

695498333

494743377

646883412

511015412

547869382

496574697

695336984

496419388

503784498

514973248

503786981

503386191

548179750

648293817

492698100

546452868

723443496

518785485

492353208

495083916

503693573

495115748

495115013

496048106

648543909

493898046

568804378

657713570

498287421

660633797

696234668

511016631

492439965

596094562

522019156

640589032

545407416

495114594

655074681

522017806

499125349

640562602

522083827

496037255

503316586

547946183

504065124

490417691

490422520

491939107

695562432

496648215

503757623

595994631

648628377

640641202

503989955

695405093

695335740

492283447

503382950

648228745

647545378

648635261

518426342

503383423

517285683

649516192

496575150

492469464

595945190

496414716

548771381

648635225

492496843

492247156

703360090

495888543

503764251

657617376

647281116

548254247

503429130

596213195

503431545

499302410

502837513

567452125

517174017

695334083

695460650

503757667

497952280

659859122

503429451

652559379

655075589

652540876

695480989

547896799

695500648

547715232

149128222

494406277

695434654

658643807

696262351

654482689

723440997

648485364

491933199

647281209

495033609

489100867

546447736

547233920

502837263

649551807

518429782

495918700

492452980

640563509

695516263

695465046

695449107

656328761

652761596

502448002

703350419

647543983

518786382

494929794

649517836

595952239

695539798

492303047

651642243

671544429

496951793

517797415

660633289

503692320

503172451

652310831

670495397

696379321

651641428

695332382

655254274

662569512

655522699

492284098

610422177

497284214

496580394

657714592

545407277

495293751

498500740

518426954

492324789

522019949

494310829

492329379

544900757

648658806

648518458

495422951

500648187

723441532

652307792

503433646

511021768

703359114

595974533

695443284

496421624

547518212

496325939

696279344

495937583

494927041

298261038

212663717

546192610

229448205

547710592

647328665

491891730

640590098

496047137

503788665

499123497

648635843

655533462

647588290

653145977

640554372

492353009

697059555

492227776

695457334

492341222

527067194

548770731

655321435

547810572

500653067

648515792

490427417

647590319

499123739

494931368

503787653

494938023

695337310

517286218

547774090

489083878

499125039

668936978

547310709

496054017

515150031

595948044

693443057

652551268

697059041

696361280

495126250

695345081

496521357

518785855

490506328

659416790

495115650

497267864

544900537

659415576

696275441

695442629

496961699

657615576

494400905

495931194

500657703

655255716

547896749

490415922

655529166

494399919

496307538

547206556

518368753

544900382

596173119

496054503

695336557

654602686

492492298

494418964

695422160

695563915

496035868

655321660

548784758

495922063

517457607

517451317

524329315

490436012

495925342

651361121

491886859

649556969

547554159

524328783

505080199

497545500

496924880

499127216

522087424

506288640

515148552

653247004

498501605

695574702

503430397

490456421

651643362

503759366

522089224

313138106

548157781

313134336

517750974

496585363

548236581

496582457

504494571

695442258

547747155

511021327

517449760

298260483

547918846

547711373

671715518

496574294

490424789

505359179

655321944

697060218

497267896

596113116

495910396

696279882

647548189

494931001

655318177

498502081

495949903

547774483

652540158

503431733

695482597

652627153

495426349

640555654

548180539

671714149

490424717

495430285

492492224

596019777

660632683

659415825

518784812

576503385

493903435

492254888

565962949

499516490

497003656

495131348

492458253

490514791

498222207

496648079

703351261

640588560

547919664

491932078

506291990

648518479

649528977

596191515

496048586

496412922

499126700

695484088

695467301

492305983

495936959

595928743

503432472

649534117

537742228

494400298

647450139

313694468

655522547

657214998

547310641

149355250

548171370

548767924

655319400

648518437

576505137

655548020

503430612

517148813

511016780

494747896

505142160

695488925

492279658

496050029

495429780

514973041

522087156

537742211

548252683

511014226

596238954

517446097

695333668

596216910

596013348

595903057

595898441

595891831

690781994

494411632

495433792

548317306

492482785

548238713

640555970

648443849

502693267

547252275

496416362

490512734

494418036

648293681

655549410

493853112

491876355

653129713

665891373

693559167

493898101

547868496

498202836

596206865

652359171

576324100

655256770

494849263

514972684

649573162

260621092

500657688

652742946

494609687

588490431

527060172

499127715

696262362

503761915

696276904

494833527

494935308

493850901

499126871

649531330

639970220

550099055

649531567

655318771

598867959

488624216

697632626

596006886

518367738

657614090

548170861

647330510

496278274

648641827

518369547

547925724

670476710

647553368

696274207

497283582

595907440

495431861

567449821

298262063

547774211

491863241

492394719

546391957

565962079

498212237

495129837

670495256

500027221

692201183

547716464

640588202

490422429

547798831

514972901

547953236

149129227

647552641

497545565

522086113

493797177

548238000

498499984

596030323

491873413

496411906

653074622

506290423

696360757

584428563

547321545

652578991

547800659

524327097

494411757

697058899

229454079

495421225

668739402

494413701

494416352

586954327

547781533

649573500

505080385

496037252

547517077

490454731

496421136

489088364

596038325

496053215

695491888

490505248

596051722

496423633

516341295

491894160

503433648

495116561

495780052

652378163

595942000

648568739

496307381

499127917

504587496

494748140

695575266

490484118

518785052

492401331

703182566

496328326

652575982

503431258

496677106

547316560

492381496

695581125

546453442

505345109

653051251

653273545

517448270

647527499

494934614

655322083

651695172

488757179

490502734

496421003

500648202

547230295

702959647

490473802

512707033

149130208

292637772

524326807

503764270

527061162

595887867

640589089

657642370

548180827

657617137

652578093

498502003

648642174

647198069

496287132

695530331

492302665

547733941

493852079

660633161

492250306

696374267

514977174

545407986

490455831

506289658

495109298

491940273

657661980

502830672

695432573

506291566

496580307

495922140

522090468

494749471

494406905

596219229

492709218

503311333

655519715

496583135

547773639

494653340

496328281

392630333

229435961

547946122

489095049

496050127

496522647

503759577

503378540

495424322

547952835

547903957

695501909

547800018

524328138

657617398

494416962

490445676

496581449

654829625

506293939

496288867

496426508

695515400

496648026

503431240

546767750

494843712

670498105

695465731

696272948

690793400

492501914

723444759

490502333

647280706

496050580

493852919

548172604

649555593

548252925

505080404

647546812

496574166

640588766

496420694

495106291

695474534

595968954

649548057

693567420

494397559

495422220

655317695

648641727

696275735

696272384

503760859

502419219

696233740

495431264

490440230

652559849

670498082

490514846

596108650

493898067

657615222

492405410

695402762

657614975

696358992

488751681

263235973

527057110

490432046

494750837

495115774

496422365

492375379

494938079

492498671

498204004

518788075

494831527

656214714

640563907

494822323

503433314

652563644

649514806

392643656

522094356

548171539

494411739

657618397

522055610

494398969

697061032

670937331

695540118

595989249

595940316

595922821

503760833

495427977

696373126

496584435

503786609

648623094

492702575

655322137

671713896

696263872

496053231

494935943

696264956

494405876

547774659

495931101

488625077

489089292

696376603

298261025

496421304

494406526

496928420

496288349

657029001

494743031

493850683

667022983

570741192

655258022

492693865

651363298

696367948

696365627

517091049

522085832

494932153

495516716

652868375

503435987

496928472

544900291

647591296

648622990

212665100

494408976

548180561

492405357

496964544

294446945

505079116

506262541

655253285

488621927

506294615

649573075

494399716

494935401

547231256

491873619

657615550

496421393

652559322

495421803

495431585

649506731

496099029

671715048

647603244

496054607

494933585

492338619

502836131

488751709

499419671

693278872

498221728

649555703

640561861

649573467

657616723

695505278

506294123

547185200

335939885

490434929

494400770

517286948

696274057

502689227

696273279

335941090

496136355

503070669

392671405

494750701

697060282

496932040

652380476

292633283

640555609

640564291

502828219

495474988

494397549

547825181

503833975

503694695

655318349

491872133

649563369

518785889

495921933

494846970

496578781

547809046

262354321

695457948

497820832

524326349

610423081

492443406

518427987

492329473

490471022

495924110

725818070

548179496

495400337

655492145

506233668

494935993

655321768

491865750

494938131

576325055

547278673

496328619

498500735

494747663

547308468

649527195

695337975

495891569

547918817

640564765

502691038

511023447

655321755

695516867

696268579

490428406

512704970

652573794

517498853

518833875

695492761

546359424

522016075

503695210

496413892

507741948

488753267

654413338

500644774

655254543

648502146

696356238

649538440

518784372

649550335

503763431

517981732

495938328

492377849

496923824

576323982

515150351

491869044

655253526

506291961

503694116

495930745

652578716

696361241

502689992

496288060

547921200

491865525

496287928

494412419

640554626

495118629

654843742

502692131

652760907

492439960

655254695

723441771

649555919

495028310

495122098

495516970

504492007

392640740

522093104

503174131

696359790

504587136

652563085

491879008

496289259

547871734

313695401

688464998

494837730

496422446

546190718

635637071

522018464

491876521

491928596

695486852

696261016

654843342

496528675

503786030

506287554

695410070

260623930

652578559

548771364

695334048

503532925

695482195

507740504

494411661

496574983

547952141

546359346

696234562

547919125

522060356

547310654

490419791

490420923

229454198

547801044

495428092

595921351

695337519

491878492

492482322

695532049

695417262

596133965

649512754

489093383

507740697

692209162

493853090

655992188

493851973

496138594

648485360

649536441

495113618

522091740

658502384

652355510

695451283

654483060

502689143

494744537

697010675

657049824

696264097

551207552

647329586

547914647

382983659

547750985

655533692

493796672

494932587

548254226

695535543

695493789

695295186

512704956

496427963

695523183

695477493

503760763

550265063

503429570

547317381

653563144

695487200

494752024

494416000

652382232

648239556

507752750

503760599

505078057

655256598

494747529

546764047

496324877

518785496

652574702

610626044

649527967

503314943

527063859

653066549

517496336

492741572

488621411

493853026

498348863

504491579

494932054

695516835

511023012

492492482

695569348

522086162

695540760

695336500

671713971

547782336

496287037

546360847

649529010

495415270

382983864

488621661

490503242

548316777

657614695

392631303

492443223

697634181

503531449

229456653

511019634

502694036

492477461

657713561

496054443

695488847

647281140

693279375

693441973

505080883

652419184

500027246

662331797

492258358

547872144

496327126

504491394

596020491

678231296

653051268

695468755

568805150

647551946

495024021

498349352

652617965

596114220

492324944

490455739

503174980

692201363

671716759

652562091

512706631

500647037

655543149

659415768

667025253

693554635

655322049

490480221

695337682

496287676

547277580

496956360

490474085

647200261

507755798

495028895

490477373

496050746

503757551

670495819

491854849

495947466

695527103

496530648

506375898

548155656

695340349

490443454

653073293

496676463

595920146

648398002

596097064

648635564

260624330

651361623

547235381

500646758

647326717

671542610

505077219

548136808

496052288

527062942

494223742

499421165

488622016

495421507

648623022

547896918

655321555

671543272

502835220

503788405

695475615

649522495

505080932

495121171

495114655

695527663

695344508

696261742

493796617

649522497

696261501

490440098

655994312

671545190

548171089

490512848

648635675

517150476

548169660

655384375

547946852

497437421

496961727

655539870

595984816

547781895

598892616

695496720

596126193

494849571

512706495

491881958

497266705

547736802

547185040

547918728

655320106

504492805

503429639

655075694

695498548

494419304

500648128

648570151

548319267

212663632

518216752

695331211

649538352

502827989

648608369

652621213

596120289

518786694

547322911

503433357

695331820

647330527

490416662

495127499

655549031

547736871

496035952

696370051

494928992

495941822

492454473

547869322

511023045

505111205

548318274

551204764

494399845

495841584

492454199

696370048

502693816

518368800

693555415

498501236

648518730

494403459

696378745

503785944

547937849

655254637

503758289

491859394

695536535

548252697

548172599

492736931

695520514

655074653

596170106

497002641

505080146

671714102

494933725

696276374

498224759

547789897

500930903

652413341

491939181

649537978

547311067

547235168

504585068

548318700

496056786

527068628

490430057

503800102

649573523

517290684

567225344

652485569

490471901

496048027

696359810

496476912

547798814

639133386

511016035

695576481

496412464

497464854

657050891

506290770

547845819

548319265

522018603

494311593

547321184

547194426

652572463

496580302

494833767

654603304

548318023

503787443

546359729

496053191

482604130

498348917

696277702

674969652

503833122

670477045

548167866

496581747

489077646

495513240

489099405

576322158

492365862

494748741

652614224

527072181

511014241

671545627

655077070

496280342

648568550

494419001

507743822

546190030

503382880

655322196

493302632

647589349

518831331

406983353

547277825

518811827

499724809

491928864

648518757

695397788

494845535

496420919

488622488

495783727

596279538

695344340

494653437

496583863

652519501

596060864

496331045

499302945

547716305

652559718

496421828

493898006

659859498

496421032

547869919

547799222

392663337

548179711

498499600

212664722

504841860

497821093

502695496

596135538

495922067

494401444

503989959

695508059

648542573

518216053

648239383

655548874

651749490

548172025

547311293

652869784

404582605

494223354

495298614

547277680

546817593

696373253

505080159

631786278

516343906

492336766

490470939

490512271

491882537

670479904

596095221

653066554

488624378

652367671

503533589

496328803

657640329

655254522

678225512

518834333

494934568

723440822

649543647

492296332

695445243

648617883

298512569

649573573

596124734

595892453

495920524

647618694

696365289

517274174

496054934

490456228

494823214

404574548

491879914

490436058

547189298

517287059

491873446

517497111

494934571

692200354

655077108

657661855

652601828

490519121

497546287

503529448

496039470

671711506

655993886

656327544

495949726

490445455

655256711

495512419

690715937

313138026

649528449

492329491

545407108

495516120

655159733

497283565

499301150

503760269

494412369

497946832

653169645

495890531

494927699

494847085

547321651

586956668

492704504

496323493

490514234

655254545

493304398

494929802

654838381

576501329

499123396

695343372

496331354

547801017

491855277

657642800

490450636

696276587

495940340

652309480

654482687

497946786

547774682

502450801

653066641

495839977

496420729

693442494

688465369

492377707

640643087

694994360

491876327

649546182

491939654

492435073

654843604

524326500

493306874

635637853

497465239

695345069

576325118

519055951

696272733

546359791

596192391

496344056

547517834

695492188

498212178

696271431

517448406

495036465

496048666

547309898

496136167

547751296

647587201

494936401

548185574

496414156

547920243

547926720

653073844

506291443

648282154

547234131

647603198

674968448

496346864

547664260

505078397

490473627

723444022

647590695

496328584

514961898

491894083

524329507

547810501

490516272

596142157

335945155

494934563

576503862

498204008

545404920

495889319

490449250

498502375

646945738

547187891

492501740

494930818

695339348

492329319

655319787

504065573

494399088

497466862

503311133

499302968

493898090

696267316

494654395

648544226

505077242

490454528

655528712

648658863

670497704

598893129

548171164

596018520

547516849

497467576

511014683

657640390

517459356

498501931

345455970

596107235

547231312

503311577

497546190

517868137

515150197

648544301

648239529

545407180

490479735

653243718

502691551

547230813

489531651

596280081

596022577

657615598

517285585

647280419

517961233

547808385

692208968

496422860

567763053

503172546

652309796

688464991

499421644

670495179

494844831

490428733

490417491

491880625

229455442

596165194

596153910

491894069

547516984

489086248

504372933

496417631

488747511

651694832

496307893

553313787

688464994

496037301

392680693

657618362

665935324

503172529

497867734

653072992

495131451

657645359

646943721

521070706

507741953

496428925

648518662

503315017

494654229

655078584

496413236

547670108

502691922

495028732

298514587

514973676

494934298

695570842

511018149

524325813

547315759

655255411

496046779

653249319

229457143

657616356

496423178

596042544

595994169

595961134

492307095

648600753

492406672

494412384

662332884

596198543

640555065

505079793

655321703

596038919

596225018

657616806

547311280

596253536

504841041

548318424

506262816

494932375

652614122

496421801

695332980

657643277

504584036

511018605

657642400

494841396

547230452

506294384

493852735

495420580

517448659

522019113

649530604

503694123

547879097

499517017

506289621

567226268

660631509

496052472

496036489

490516012

496042794

649506609

659859605

500648146

656270601

503785246

506292294

547185339

548146288

586956687

647552090

495428144

495429496

495111317

652508911

263252753

596213561

648519419

647329794

492427028

491859617

496476870

313138156

497546347

652551567

524329545

505078744

649538285

495921823

488625235

682032431

652356955

504843034

648617316

494836839

496288939

490446595

518367717

703350985

584427923

502690350

596192498

488625784

499515897

655320290

498500544

505146288

490450994

651695092

693572209

547194542

547322117

511019960

693572525

647527632

652354133

492400862

317385106

497865056

640562149

494928748

495931733

263255708

631784712

652540747

500027884

690788919

492708547

595940404

548218775

522019955

548172620

544900667

553312991

653563549

651640104

546452900

697633681

496328544

527066264

596230204

335941718

586955696

496579957

544899729

495106981

505344606

546830783

697634451

647552851

498501232

548317388

584441246

263236950

652539145

652741042

652137017

496328248

546331739

496961684

694993208

639135989

594833870

498106176

518367958

490444170

492401727

496286066

500644248

649543926

496330464

671546542

492503664

547746151

503377883

387775607

654413067

690799686

496329010

676330297

494746206

653066918

490426000

522020739

547288818

492394735

547288612

596176644

494931646

517450496

498500849

488758677

595954514

492453049

660631000

547233503

503436188

496921901

660630950

345456198

697633199

547278518

527064059

490435181

657617803

547925263

494399042

547332241

648229016

503385587

500646276

547800045

494401595

649555269

545407540

494412417

517460414

649546274

696379325

695342269

507745041

547233388

692199679

496476188

576325170

547789441

498499854

651640271

548318657

502829720

665898507

495925675

494751588

522014946

518785943

595894667

649555685

649545922

648518897

647330460

493852008

491891778

639136627

298515544

511013797

547188791

335943162

595924946

680466751

547920267

653275594

502455395

548172593

494835519

492741696

652604225

547800986

495115039

651652765

511018120

690769534

496959534

490441430

506291378

494749294

494926932

502951802

517274018

649556302

660630959

490422661

492497941

518833512

490439774

511021778

494418969

490427293

511022683

660632799

546360039

494929003

495116431

692212699

503787880

490451567

655254406

313134231

649534015

503399709

676327993

695339336

671713685

496414958

494848505

229450631

650331512

654482111

665397704

596028845

506262128

547844092

547306744

502690551

690797321

547667248

547809309

511019040

511017771

680463362

649538658

670495228

515147926

649555932

545310169

496954348

669227866

545433179

655522395

647280512

494398716

503218223

657617740

505345498

495123451

695423867

490432296

695416078

576321069

697060918

497265117

524328922

514972919

498499148

576321171

653051688

649527170

696275492

647551959

695342779

631783742

494838011

494047380

495929844

547773981

651360975

657214119

696278449

495425966

504494841

696276135

595960474

649558188

547642673

495933828

547782243

657618030

490451540

596033913

490471210

511012602

494653508

494934609

700231335

665398419

547798933

527072331

495301248

703184784

496346508

640642390

521070666

495295807

313135009

408473026

490503169

494412498

404584504

495119170

647329664

547231644

649574152

494402272

695436719

595937378

676327506

494932727

647607848

596147213

547800187

492321200

696276185

517286952

527062458

567224405

547199514

495431554

522090689

596120466

498222546

695346128

496959901

654843360

690714358

548153978

500648959

490706383

496050282

392623345

547642643

696377234

511016036

495114238

495130652

229455439

500657662

610421618

657615004

548172335

651693448

655318529

648543923

586954459

653239272

502693436

496584016

693568111

517173169

680466782

690792243

516802091

494412841

522090474

697985952

503433553

646943499

498212369

488625188

652574764

495031476

547823701

545406155

657218184

496043339

570741210

547307991

696377466

489085928

652539181

505079280

543403683

494749339

547868853

496415176

517443567

671542205

499421128

496136816

696372869

696363997

697092033

514973669

503692548

649545571

503173722

496049936

649545743

648635406

498499457

500644781

494934836

652418328

649528729

492724202

647280917

493902041

495841451

671710782

696366990

496289117

495929122

317387726

655528567

494744260

696371058

647280431

651362081

692213315

495127492

292630980

495427990

696266824

671546823

653246896

671716761

671549676

492704030

655077901

595916332

547323161

495925970

652305601

496056695

495107191

495429638

648570122

567762504

495886853

663481755

663471253

503431442

651774655

493852973

503762405

497867477

492483036

671712227

652357744

490434118

493902051

695333287

490440030

503529843

651642360

495942439

640550103

547930089

504373064

490498050

665961141

703350429

263237287

690799488

657616553

695524917

663458781

655257389

518783373

506292226

490418005

680467057

506291918

595887964

648570423

670497768

517149338

697057025
