# Supplementary material for: Structure and sequence analyses of Bacteroides proteins BVU_4064 and BF1687 reveal presence of two novel predominantly-beta domains, predicted to be involved in lipid and cell surface interactions
Source: BMC Bioinformatics. 2015 Jan 16;16(1):7. doi: 10.1186/s12859-014-0434-7 (PMC4387736; doi:10.1186/s12859-014-0434-7)
Supplement: Additional file 3: — Table S1. Crystallographic data and refinement statistics for the protein BVU_4064 (PDB code 3kog). Values in parentheses are for the highest resolution shell. Table S2. Crystallographic data and refinement statistics for the protein BF1687 (PDB code 3g3l). Values in parentheses are for the highest resolution shell. [file 12859_2014_434_MOESM3_ESM.docx]

**Table S1.**

Crystallographic data and refinement statistics for the protein BVU_4064 (PDB code 3kog). Values in parentheses are for the highest resolution shell.

|  | **λ_1_ MAD-Se** |  |  |
| --- | --- | --- | --- |
| **Data collection** | | | |
| Space group | I 2_1_ 2_1_ 2_1_ | | |
| Unit cell parameters (Å) | a = 66.80, b = 70.34, c = 104.10 | | |
| Wavelength (Å) | 0.85503 | 0.97926 | 0.97845 |
| Resolution range (Å) | 29.814-1.850 (1.90-1.85) |  |  |
| No. of observations | 86988 |  |  |
| No. of unique reflections | 21313 |  |  |
| Completeness (%) | 100.0 (100.0) |  |  |
| Mean *I/σ (I)* | 10.2 (1.0) |  |  |
| *R_merge_* on *I*^†^ (%) | 9.1 (72.6) |  |  |
| *R_meas_* on *I*^‡^ (%) |  |  |  |
| **Model and refinement statistics** | | | |
| Resolution range (Å) | 29.81-1.85 |  |  |
| No. of reflections (total) | 21310^§^ |  |  |
| No. of reflections (test) | 1095 |  |  |
| Completeness (%) | 100.0 |  |  |
| Data set used in refinement | λ_1_ |  |  |
| Cutoff criteria | \|F\|>0 |  |  |
| *R_cryst_*^¶^ | 0.182 |  |  |
| *R_free_*^¶^ | 0.214 |  |  |
| **Stereochemical parameters** | | | |
| Restraints (RMSD observed) |  |  |  |
| Bond angles (º) | 1.621 |  |  |
| Bond lengths (Å) | 0.018 |  |  |
| Average isotropic *B* value^††^ (Å^2^) | 18.4 |  |  |
| ESU^‡‡^ based on *R_free_* (Å) | 0.129 |  |  |
| Protein residues/atoms | 226 / 1729 |  |  |
| Waters/solvent molecules | 169 |  |  |
|  | | | |
| ^†^ *R_merge_* = Σ*_hkl_*Σ*_i_*\|*I_i_(hkl) - (I(hkl))*\|/Σ*_hkl_* Σ*_i_(hkl)*.  ^‡^ *R_meas_* = Σ*_hkl_*[*N/(N*-1)]^1/2^Σ*_i_*\|*I_i_(hkl) - (I(hkl))\|/*Σ*_hkl_*Σ*_i_I_i_(hkl)* (Diederichs & Karplus, 1997).  ^§^ Typically, the number of unique reflections used in refinement is slightly less than the total number that were integrated and scaled. Reflections are excluded owing to negative intensities and rounding errors in the resolution limits and unit-cell parameters.  ^¶^ *R_cryst_* = Σ*_hkl_*\|\|*F*_obs_\| - \|*F*_calc_\|\|/Σ*_hkl_*\|*F*_obs_\|, where *F*_calc_ and *F*_obs_ are the calculated and observed structure-factor amplitudes, respectively. *R_free_* is the same as *R_cryst_* but for 4.9% of the total reflections chosen at random and omitted from refinement.  ^††^ This value represents the total *B* that includes TLS and residual *B* components.  ^‡‡^ Estimated overall coordinate error (Collaborative Computational Project, Number 4, 1994; Cruickshank, 1999). | | | |

**Table S2.**

Crystallographic data and refinement statistics for the protein BF1687 (PDB code 3g3l). Values in parentheses are for the highest resolution shell.

|  | |
| --- | --- |
|  | **λ_1_ MAD-Se** |
| **Data collection** | |
| Space group | P 3_2_ 2 1 |
| Unit cell parameters (Å) | a = 107.62, b = 107.62, c = 89.30 |
| Wavelength (Å) | 0.97966 |
| Resolution range (Å) | 29.761-2.200 (2.26-2.20) |
| No. of observations | 227775 |
| No. of unique reflections | 30727 |
| Completeness (%) | 100.0 (100.0) |
| Mean *I/σ (I)* | 11.8 (2.2) |
| *R_merge_* on *I*^†^ (%) | 13.4 (93.1) |
| *R_meas_* on *I*^‡^ (%) | 14.5 (100.1) |
| **Model and refinement statistics** | |
| Resolution range (Å) | 29.76-2.20 |
| No. of reflections (total) | 30702^§^ |
| No. of reflections (test) | 1548 |
| Completeness (%) | 99.9 |
| Data set used in refinement | λ_1_ |
| Cutoff criteria | \|F\|>0 |
| *R_cryst_*^¶^ | 0.170 |
| *R_free_*^¶^ | 0.206 |
| **Stereochemical parameters** | |
| Restraints (RMSD observed) |  |
| Bond angles (º) | 1.745 |
| Bond lengths (Å) | 0.016 |
| Average isotropic *B* value^††^ (Å^2^) | 45.230 |
| ESU^‡‡^ based on *R_free_* (Å) | 0.134 |
| Protein residues/atoms | 291 / 2170 |
| Waters/solvent molecules | 207 |
|  | |
| ^†^ *R_merge_* = Σ*_hkl_*Σ*_i_*\|*I_i_(hkl) - (I(hkl))*\|/Σ*_hkl_* Σ*_i_(hkl)*.  ^‡^ *R_meas_* = Σ*_hkl_*[*N/(N*-1)]^1/2^Σ*_i_*\|*I_i_(hkl) - (I(hkl))\|/*Σ*_hkl_*Σ*_i_I_i_(hkl)* (Diederichs & Karplus, 1997).  ^§^ Typically, the number of unique reflections used in refinement is slightly less than the total number that were integrated and scaled. Reflections are excluded owing to negative intensities and rounding errors in the resolution limits and unit-cell parameters.  ^¶^ *R_cryst_* = Σ*_hkl_*\|\|*F*_obs_\| - \|*F*_calc_\|\|/Σ*_hkl_*\|*F*_obs_\|, where *F*_calc_ and *F*_obs_ are the calculated and observed structure-factor amplitudes, respectively. *R_free_* is the same as *R_cryst_* but for 4.9% of the total reflections chosen at random and omitted from refinement.  ^††^ This value represents the total *B* that includes TLS and residual *B* components.  ^‡‡^ Estimated overall coordinate error (Collaborative Computational Project, Number 4, 1994; Cruickshank, 1999). | |
